# A cortical edge-integration model of object-based lightness computation that explains effects of spatial context and individual differences

**DOI:** 10.3389/fnhum.2014.00640

**Published:** 2014-08-22

**Authors:** Michael E. Rudd

**Affiliations:** ^1^Howard Hughes Medical Institute, University of WashingtonSeattle, WA, USA; ^2^Department of Physiology and Biophysics, University of WashingtonSeattle, WA, USA

**Keywords:** lightness, brightness, achromatic color, cortical ventral stream, neural computation

## Abstract

Previous work has demonstrated that perceived surface reflectance (lightness) can be modeled in simple contexts in a quantitatively exact way by assuming that the visual system first extracts information about local, directed steps in log luminance, then spatially integrates these steps along paths through the image to compute lightness (Rudd and Zemach, 2004, 2005, 2007). This method of computing lightness is called edge integration. Recent evidence (Rudd, 2013) suggests that human vision employs a default strategy to integrate luminance steps only along paths from a common background region to the targets whose lightness is computed. This implies a role for gestalt grouping in edge-based lightness computation. Rudd (2010) further showed the perceptual weights applied to edges in lightness computation can be influenced by the observer's interpretation of luminance steps as resulting from either spatial variation in surface reflectance or illumination. This implies a role for top-down factors in any edge-based model of lightness (Rudd and Zemach, 2005). Here, I show how the separate influences of grouping and attention on lightness can be modeled in tandem by a cortical mechanism that first employs top-down signals to spatially select regions of interest for lightness computation. An object-based network computation, involving neurons that code for border-ownership, then automatically sets the neural gains applied to edge signals surviving the earlier spatial selection stage. Only the borders that survive both processing stages are spatially integrated to compute lightness. The model assumptions are consistent with those of the cortical lightness model presented earlier by Rudd (2010, 2013), and with neurophysiological data indicating extraction of local edge information in V1, network computations to establish figure-ground relations and border ownership in V2, and edge integration to encode lightness and darkness signals in V4.

## Introduction

**Figure 1:**
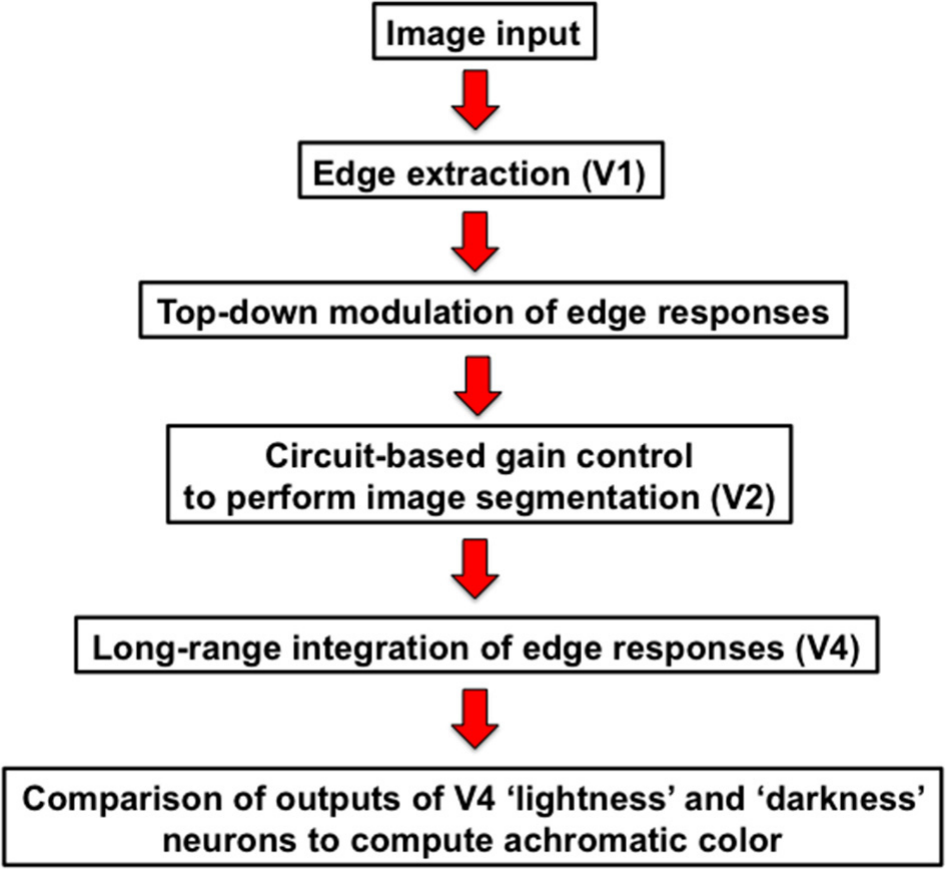
**Cortical stages of lightness computation in the neural edge integration model**.

In this paper, I outline a theory of object-based neural lightness computation occurring within the ventral stream of visual cortex (Figure 1) and apply this theory to problems of gestalt grouping and individual differences in lightness perception. The theory includes bottom-up, top-down, and mid-level computations that are identified respectively with: (1) early sensory encoding of local oriented contrast occurring along the pathway from retina to V1; (2) task-specific top-down cortical feedback modulation of the early neural contrast code in V1; and (3) neural circuit computations in V2 that perform functions related to image segmentation. A neural representation of surface reflectance (“lightness” for short) is constructed by a long-range spatial integration of the cortical responses to oriented contrast, or edges, whose neural gains are adjusted prior to spatial integration by the top-down and mid-level computations occurring in cortical areas V1 and V2. The long-range edge integration is proposed to occur in area V4. The achromatic colors assigned to each image location are computed by a mechanism that compares the output of the long-range integrator neurons in V4.

I will begin by discussing a simplified version of the model and show how this model accounts for classical and recent data on effects of spatial context on lightness, including the dependence of lightness induction on distance and differences in induction strength for increments and decrements. The model produces lightness filling-in phenomena as a byproduct of edge integration. After presenting this simplified, or “basic,” model, I explain the need to incorporate additional computations related to attentional selection and image segmentation. I show how the elaborated model accounts for individual differences in the staircase-Gelb lightness paradigm. I close by discussing some outstanding problems for the theory.

## The basic neural edge-integration model

One of the simplest of all lightness illusions is *simultaneous contrast*: a mid-gray paper viewed in the context of a black surround looks lighter than the same paper viewed in the context of a white surround (Chevreul, 1839/1967). Simultaneous contrast has been studied for 175 years, yet there are still disagreements regarding its correct interpretation. One common explanation is that the visual system does not care about luminance *per se*, but rather about luminance ratios, or *relative* luminance. In fact, early visual neurons in the retina and cortex do recode pointwise image luminance into a local contrast code. At sufficiently high light levels, the retinal output is proportional to the local Weber contrast Δ*I/I*. Thus, cortical mechanisms tasked with perceiving reflectance of the gray papers in the simultaneous contrast display receive a neural signal telling them that the intensity of the gray paper differs from the surround intensity by a given fraction of the surround intensity. Retinal ON cells relay information about the relative luminances of image regions whose luminances are higher than that of the surround; whereas, OFF cells relay information about the relative luminances of regions whose luminances are lower than that of the surround.

Wallach (1963) found that human observers, when asked to match in appearance two dark disk targets surrounded by annuli of higher luminance, set the disk/annulus luminance ratios to be approximately equal for a match. This is mathematically equivalent to setting the local Weber fraction at the disk/annulus edges equal in order to achieve an appearance match. Wallach's experiments were performed around the same time that neurophysiological evidence for Weber fractional neural encoding by ON cells and OFF cells was discovered (Kuffler, 1953) and Wallach suggested that his observers' ratio matches might be explained on the basis of this finding.

Further support for this idea comes from data showing that the appearance of a region of homogeneous luminance is strongly influenced by the luminance contrast at the region's border. If appearance is determined solely by neural activity at the edge, then an additional neural mechanism is needed to reconstruct—or “fill in”—regional appearance on the basis of edge contrast. I return to this point below and propose that “filling-in” of surface percepts in natural vision is related to perceptual illusions of color spreading from borders, such as the Craik-O'Brien-Cornsweet (O'Brien, 1958; Craik, 1966; Cornsweet, 1970) and Watercolor (Pinna et al., 2001) illusions.

To build a quantitative theory of lightness filling in from edges, one has to consider the fact that a large asymmetry exists in the strength of the induction effect produced by the size of the luminance step at the edge, depending on the contrast polarity of the border: that is, whether the region is light inside, or dark inside, relative to the surround. A lighter surround has a very great impact on the appearance of a dark region, while a darker surround has a much smaller impact on the appearance of a light region (Heinemann, 1972; Rudd and Zemach, 2004, 2005, 2007; Vladusich, 2013a,b). Gilchrist (1988) measured the relative influences of dark and light surrounds on identical gray papers in a simultaneous contrast display and concluded that most of the illusion was accounted for by darkness induction from the light surround to the decremental paper.

To account for this marked asymmetry in the relative strengths of lightness and darkness induction, Wallach—and later Gilchrist—proposed a perceptual principle, known as “highest luminance anchoring” which asserts that any surface having the highest luminance among a group of commonly-illuminated surfaces will be perceived as white. Furthermore, the lightnesses of all other surfaces appearing within the group of commonly illuminated surfaces are determined by the surface's luminance ratio with respect to the white point (Wallach, 1963; Cataliotti and Gilchrist, 1995; Gilchrist et al., 1999; Gilchrist, 2006; Gilchrist and Radonjić, 2010). Importantly, the highest luminance rule predicts a very strong asymmetry between the strengths of lightness and darkness induction when the target and surround are the only surfaces appearing within an illumination framework. In that case, the highest luminance rule predicts a complete *absence* of a surround effect for incremental targets because the target itself is then the highest luminance within the illumination framework containing only the target and surround.

**Figure 2:**
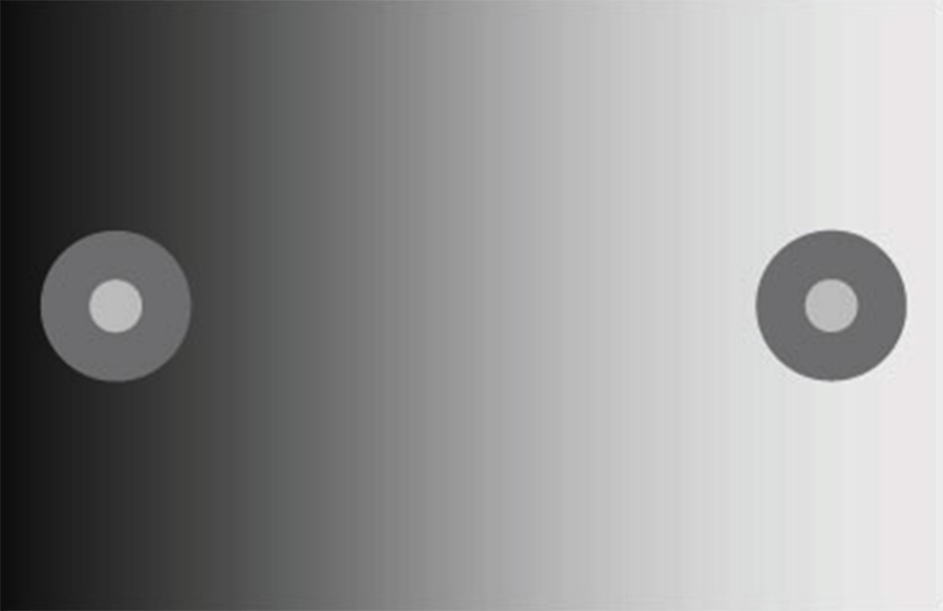
**Perceptual edge integration demo**. The disks and annuli on the two sides of the display have the same luminance, so the luminance ratio at the disk/annulus edge is the same on the two sides. The shallow luminance gradient across the background field produces outer annulus edge ratios of opposite sign on the two sides of the display. The outer annulus edge on the right signals to the brain that the annulus is darker than the background field; whereas, the outer edge on the left signals that the annulus is lighter than the background field. The key observation is that the polarity of this edge influences the appearance of both the annulus and the disk that is embedded inside it. The disk appearance cannot be explained by a filling-in mechanism that diffuses from the outer annulus border and is stopped by the disk/annulus edge. The filling-in signal must be able to reach the region of the disk. This fact is explained in the model by the mechanism of cortical edge integration.

While anchoring theory emphasizes the grouping of surfaces for purposes of lightness computation by regions of common illumination, it ignores the perceptual evidence for other grouping principles in lightness (Bressan, 2006). In particular, it ignores a large body of literature suggesting that the influence of spatial context on lightness declines with distance up to about 10 deg of visual angle (Diamond, 1953; Reid and Shapley, 1988; Rudd and Zemach, 2004) and, thus, that some type of grouping-by-proximity rule contributes to lightness perception. My colleagues and I have shown that lightness computation in simple contexts, including disk-annulus and simultaneous contrast displays, can be characterized in a *quantitatively exact* way by a mathematical theory in which weighted sums of luminance steps at edges—with the steps measured in units of log luminance—are summed in the direction of the target surrounding over a spatial window of about 10° (Rudd, 2001, 2003, 2010, 2013; Rudd and Arrington, 2001; Rudd and Popa, 2007; Rudd and Zemach, 2004, 2005, 2007). In what follows, I will refer to this theory as *edge integration theory*, and to the more speculative neural theory proposed here to account for the mathematical properties of edge integration as *neural edge integration theory*. A demonstration of the perceptual phenomenon of edge integration is shown in Figure 2.

From the mathematical standpoint, edge integration can be equivalently described as a process that produces lightness values from a weighted sum of logarithms of luminances ratios (hereafter, *log luminance ratios*) at edges. The log luminance ratio, or $log\frac{L_{n\;+\;1}}{L_{n}}$ (where the subscripts *n* and *n* + 1 denote the sequence of luminances encountered along the path over which the log luminance ratios at edges are spatially summed) is closely related both to the *logarithm* of the Weber fraction at the edge—because the luminance ratio is just the Weber fraction plus 1—and to the logarithm of local Michelson contrast (Reid and Shapley, 1988). A weighted sum of log luminance ratios is also closely related to the (unweighted) sum of log luminance ratios that determines lightness in Land's retinex theory of color constancy (Land and McCann, 1971; Land, 1977; Rudd, 2010, 2013). In fact, edge integration theory can be thought of as a modified version of retinex, in which the perceptual weights assigned to contextual edges for the purpose of computing lightness depend on distance.

An additional factor, other than distance, that has been demonstrated to influence the weight given to a particular contextual edge in determining the lightness of a target is the edge contrast polarity, defined here as whether the dark or light side of the edge points in the direction of the target whose lightness is being computed (Rudd and Zemach, 2004, 2005, 2007; Rudd and Popa, 2007; Vladusich et al., 2006a; Rudd, 2010, 2013). Whereas, anchoring theory asserts that there should be a strong contrast induction from the surround luminance on the lightness of a decremental target, but no contrast induction from the surround on the lightness of an incremental target (Gilchrist et al., 1999; Gilchrist, 2006), edge integration theory asserts that the difference in the relative strengths of lightness and darkness induction is one of degree, rather than all-or-none (Rudd and Zemach, 2004, 2005, 2007; Rudd, 2010, 2013), and can be explained as a results of the different perceptual weights assigned to edges depending on the edge polarity. Overall, the evidence suggests that weights associated with edges whose light side points toward the target are only about 1/3 as large as weights associated with edge whose dark sides point toward the target, after the effects of distance have been taken into account (Rudd, 2013).

The most “basic” edge integration model imaginable would therefore combine the fact that different perceptual weights are given to edges of opposite contrast polarities with the fact that these edge weights also tend to fall off monotonically with distance from the target. I will refer to the “basic” model as one that incorporates distance and contrast polarity as independent factors to determine the total weight associated with an edge in computing a target's lightness.

Rudd (2013) proposed that the polarity dependence of the edge weights may arise from separate power law transformations of the neural Weber ratio responses to incremental and decremental luminance occurring early in visual processing, such that ON cell responses to incremental luminance are proportional to the cube-root of incremental intensity, while OFF cell responses to decremental luminance are linearly proportional to decremental intensity. The compressive nonlinearity that applies to ON responses only could originate as early as the cone photoreceptors, which exhibit a rapid compressive adaptation when exposed to light and a release from this compressive adaptation when subsequently exposed to darkness (Angueyra and Rieke, 2013). An early cube-root ON cell response to incremental luminance would also explain a host of other visual phenomena, including Stevens' brightness law, according to which brightness varies in proportion to the cube root of luminance (Stevens, 1975), and the fact that simple reaction time (Pieron, 1914; Luce, 1986) and critical duration (Raab, 1962; Rudd, 1996) for incremental luminance both vary *inversely* with the cube root of luminance. If this hypothesized power law stage of sensory encoding that gives rise to these laws was followed at a subsequent stage of neural processing by a logarithmic transformation, the 1/3 power law exponent that applies to incremental intensity at the early encoding stage would be converted into a gain factor of 1/3 that would *multiply* the neural response to increments at the subsequent, post-log-transform stage. Furthermore, if the neural response to decremental luminance at the early stage was *linear*, as hypothesized, the gain factor applying to decremental luminance at the post-log-transform stage would be 1 in the case of decrements. Then the neural “weight” associated with incremental luminance steps would then be 1/3 the size of the weight associated with decremental steps. Rudd (2013) discussed how this differential weighting could be realized in neural processing.

The hypothesis that the initial sensory response to incremental and decremental luminance is subject to a logarithmic transformation at a later stage of neural processing is consistent with a number of pieces of evidence, both of a neural and perceptual nature. Simple cells in cortical area V1, which are often modeled as linear spatial filters followed by a threshold nonlinearity—have also been alternatively modeled as having a linear dependence on *log luminance* (Kinoshita and Komatsu, 2001; Vladusich et al., 2006b), or log contrast (Tolhurst et al., 1983, their Figure 1). This suggests that the hypothesized logarithmic transformation might occur prior to the simple cell stage in V1. A proportional neural response to log contrast at the cortical level would also help to explain the findings of Whittle (1994) that the *brightness* (perceived intensity) of both incremental and decremental targets is described by a function of the log-transformed Weber fraction for targets whose luminances are sufficiently close to that of the surround. The brightness of higher contrast targets is proportional to log of the target's luminance ratio with respect to the surround (Whittle, 1992). However, recent evidence from an fMRI imaging study alternatively suggests that the cube-root compressive nonlinearity is maintained at least up to the level of V1 (Kay et al., 2013). So the particular neural stage as which the logarithmic transformation occurs is still an open question. From the standpoint of the computational lightness model, what is important is that the 1/3 power law compression applies only to increments, while the response to decrements is linear, and that a logarithm transformation occurs at some cortical stage to convert the respective power law exponents corresponding to increments and decrements to gain factors that multiply a neural response that is proportion to steps in log luminance.

**Figure 3:**
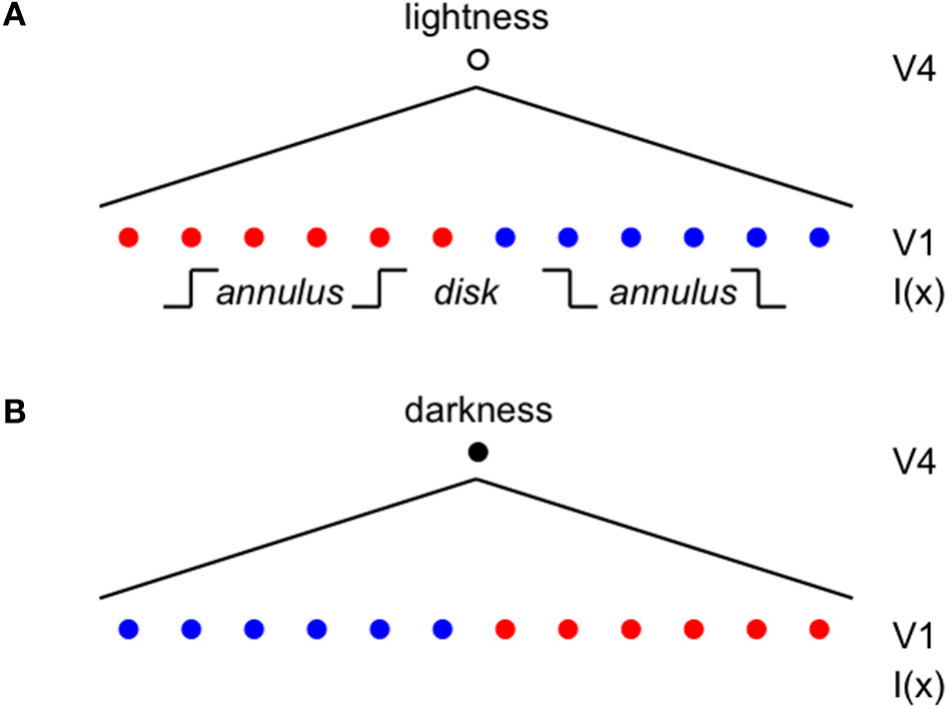
**Cortical architecture of the basic edge integration model showing its response to an incremental disk-annulus display. (A)** Lightness neurons in V4 integrate the outputs of simple cells in V1 having asymmetric receptive fields that detect edges whose light sides point toward the lightness neuron's receptive field center. The red dots indicate the positions of V1 neurons that respond to edges having higher luminance on the left side; and the blue dots indicate the positions of V1 neurons that respond to edges having higher luminance on the right side. The diagonal lines fanning downward from layer V4 to layer V1 schematically represent the V4 receptive fields that spatially integrate the outputs of V1 neurons to edges of appropriate contrast polarity in order to activate separate “lightness” and “darkness” units in V4 signaling the magnitudes of lightness and darkness at the location of the V4 receptive field centers. The linear receptive field profile should not be taken literally; the actual profile is unknown. In fact, the profiles is more likely to have the form of a decaying exponential because edge integration models that have been applied to disk-annulus stimuli suggest that edge weights fall off linearly with the logarithmic of distance to good approximation from the target (Rudd and Zemach, 2004). I(x) indicates the locations within the lightness neuron's receptive field of the inner and outer annulus edges of a “double-increment” disk-annulus display, that is a display whose inner and outer annulus edges are both of the light-inside contrast polarity type, such as the one illustrated on the left side of Figure 2 (Rudd and Zemach, 2007). The labels “disk” and “annulus” indicate the locations of the disk and annulus regions in a one-dimensional cross-section of the disk-annulus display. **(B)** Darkness neurons in V4 similarly integrate the outputs of simple cells having asymmetric receptive fields that detect edges whose dark sides point toward the darkness neuron's receptive field center. A double-increment disk annulus display contains no edges that stimulate a darkness neuron. In the most general case, some of the edges in a lightness display will excite lightness neurons, and some will excite darkness neurons. The achromatic color assigned to a given location in the image will depend on the difference in the excitation levels of the V4 lightness and darkness neurons whose receptive fields are centered on that location (Rudd, 2010).

Once the incremental and decremental responses have been neurally recoded in terms of log luminance, then all that is required to account for edge integration in lightness perception is a long-range spatial integration mechanism that appropriately sums responses to steps in log luminance at edges. A long-range mechanism that sums log-transformed neural responses to edges whose dark sides point toward the receptive field center of the long-range mechanism would compute an integrated darkness signal. A long-range mechanism that sums log-transformed neural responses to edges whose light sides point toward the receptive field center of the long-range mechanism would similarly compute an integrated lightness signal. Separate subpopulations of neurons in V4 having at least some of the properties required to instantiate these separate long-range lightness and darkness computations have recently been identified (Bushnell et al., 2011). These neurons receive input from V1 both directly and through V2, have sufficiently large receptive fields to account for the spatial range of contextual effects in lightness, and respond only to stimuli that are either luminance increments or decrements, respectively. However, the key question of whether these neurons actually sum directed steps in log luminance across space has not yet been examined. Other neural subpopulations in V4 respond only to chromatic, rather than achromatic, spatial contrast, suggesting that one function of V4 may be to instantiate an edge integration process that accounts for the effects of spatial context on color, more generally. If V4 neurons do perform edge integration in separate “lightness” and “darkness” channels, then the perceived achromatic color at a given spatial location within the input image could be computed from the relative responses of the lightness and darkness neurons having receptive fields centered on the specified location (Rudd, 2010, 2013). Presumably, this would occur beyond V4, mostly likely in area TEO or TE: areas that mark the end of the line within the ventral stream and contain representations of objects and surfaces that can be accessed by consciousness and stored in memory through interactions with entorhinal cortex (Murray et al., 2007). Figure 3 illustrates the way in which the lightness of the incremental disk-annulus stimulus shown on the left side of Figure 2 would be computed by the cortical edge integration theory described to this point and provides a schematic representation of the cortical edge integration theory, more generally.

Clearly, additional neural machinery would be required to give a full account of lightness perception, since nothing has been said so far here about perceived illumination, figural organization scission, or the three-dimensional world, to name but a few other factors that are known to influence lightness. But the model described to this point includes the most basic neural properties required to explain edge integration phenomena in lightness. In what follows, I will show how lightness judgments in somewhat more complex perceptual paradigms can be modeled by further elaborating this basic model to include additional neural mechanisms that perform mid- and high-level computations related to image segmentation and attention. My goal here is not to present a complete model of lightness computation, but rather to sketch out a skeletal model of a cortical circuit by which lightness might be computed in the ventral stream of visual cortex, starting with edges and ending with representations of surfaces and objects.

## Lightness filling-in as a byproduct of edge integration

An important implication of the model shown in Figure 3 is that it predicts that percepts of lightness and darkness should appear to radiate spatially from the light and dark sides of luminance steps. The model exhibits this behavior because the model lightness and darkness neurons in V4 are activated by edges that can, in general, be a considerable distance away from the receptive field center of the V4 neuron. The receptive field center is assumed be the point to which the spatially integrated lightness or darkness value computed by the neuron is assigned in the overall computation of achromatic color.

**Figure 4:**
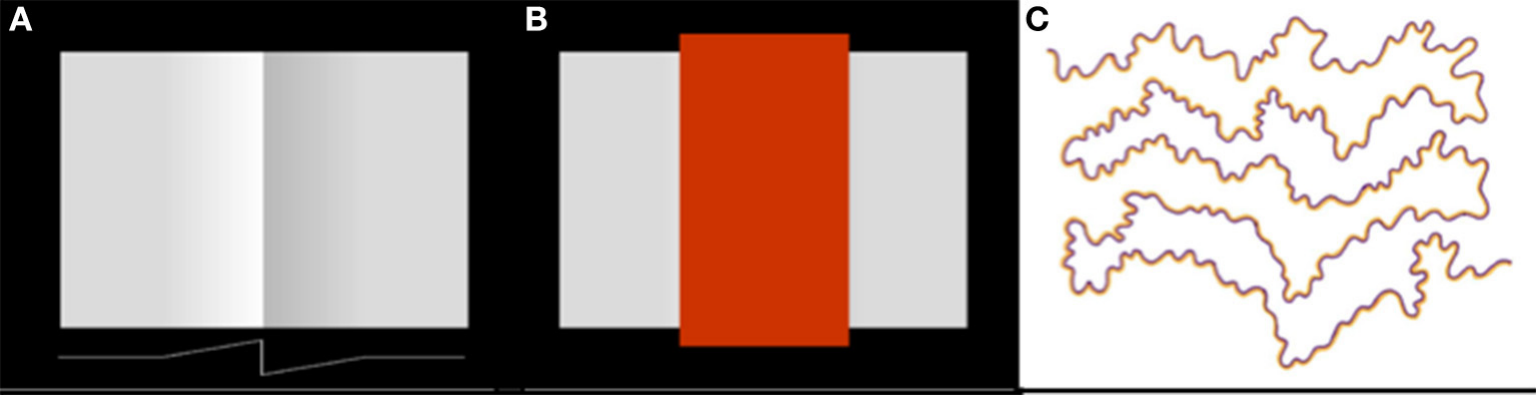
**Examples of lightness and color filling-in phenomena. (A)** Craik-O'Brien-Cornsweet illusion. The vertical luminance edge at the center of the figure generates a lightness illusion that appears to fill in the regions on either side of the edge, filling in the region on the dark side of the edge with darkness and the region on the light side of the edge with lightness. A one-dimensional horizontal cross-section of the actual luminance profile of the perceptually filled-in region is shown at the bottom of the panel. **(B)** Same as **(A)** except with the region of the vertical edge masked out. **(C)** Watercolor illusion. The sinuous high-contrast chromatic edge creates an illusory percept of filled-in color within the regions lying between the edges, whose tints are consistent with the colors of the regional borders.

The exact spatial distribution of the radiating patterns of lightness and darkness will depend on the shape of the lightness and darkness neuronal receptive fields. In Figure 3, I have illustrated the receptive field weighting function as a linearly decreasing function of distance from the receptive field center. But there is reason to believe—on the basis of psychophysical data concerning the spatial extent of lightness induction—that the actual spatial profile of the receptive field may a decaying exponential or some other function that decreases rapidly near the receptive field center and more slowly further from the center (Stevens, 1967; Rudd and Zemach, 2004). The reader may get some intuition for the rate of falloff by carefully examining the light and dark colors that appear to spread from either side of the central vertical edge in Figure 4A.

Whatever the profile of the spatial decay of lightness and darkness induction signals from edges, the model provides at least a partial explanation of the achromatic color filling-in that occurs in illusions like the Craik-O-Brien-Cornsweet effect (Figures 4A,B); and—by extension to the chromatic domain—other color filling-in phenomena, such as the Watercolor effect (Figure 4C). In filling-in phenomena, a false color is induced by the contrast at a regional border and appears to fill the region lying between borders. Neural mechanisms that have previously been proposed to account for perceptual filling-in include low spatial frequency channels whose filter scales span the spatial extent of the region between the borders (Blakeslee and McCourt, 1997) and diffusive neural filling-in signals that spread dynamically from borders to fill in regions lying between borders in a topographical neural map of the visual environment (Grossberg and Mingolla, 1985). In diffusive filling-in models, the spreading color signal induced by a border is stopped when it encounters the next border in the topological map, which blocks the diffusing signal.

An argument against the idea that color filling-in phenomena result from the activation of a low spatial frequency visual channel is that the spatial extent of filling-in is larger than the distance spanned by the lowest spatial frequency filters in human vision, which are centered on a frequency of about 0.5 cycle/deg and have a bandwidth of about one octave (Wilson and Gelb, 1984). A low spatial frequency channel could therefore account for color spreading over a range of a few degrees, at most. But the Watercolor effect has been shown to spread perceptually over distances as large as 45° (Pinna et al., 2001), a spatial range that is about one order of magnitude larger that the range that could be explained by low spatial frequency filtering of the input image.

An argument against the second, diffusive filling-in, explanation of perceptual filling-in is that the colors generated by the borders in Figure 4C do not appear to leak from the open ends of the perceptually filled-in regions, even though there is no border there to stop the spreading color signals. Another argument against diffusive filling-in—one that is of prime importance in the current context—is that filling-in signals that are stopped at borders cannot provide a basis for explaining the fact of perceptual edge integration (Rudd, 2010). The achromatic colors of the disks in Figure 2 depend not only on the luminance step at the edge between the disk and annulus, but also on the luminance step at the edge between the annulus and the background. To account for both of these influences with an edge-based theory of color induction requires a mechanism that can sum the influences of edges located at multiple distances from the target. For this to happen, it cannot be the case that an edge induction effect produced by the more distant edge—in Figure 2, the disk/annulus edge—is blocked by a border that lies closer to the target—in Figure 2, the disk border itself. The hierarchical edge integration model shown in Figure 3 provides a parsimonious explanation of perceptual filling-in in that it can account for both filling-in and edge integration with one mechanism.

**Figure 5:**
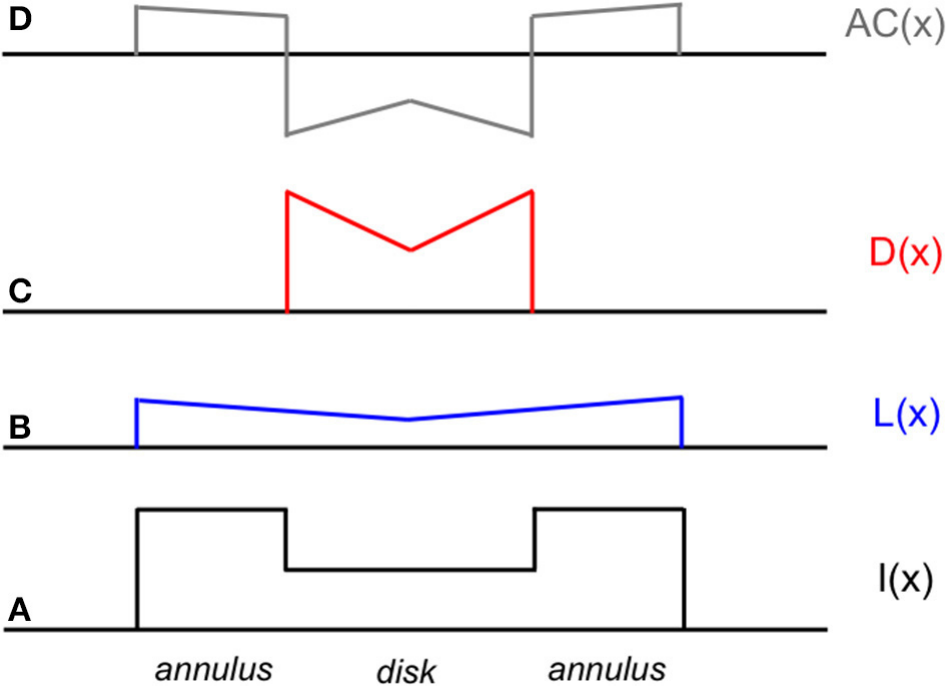
**Schematic diagram of neural activations at various stages of the cortical lightness computation model in response to a decremental disk-annulus stimulus**. From bottom to top (following the convention of Figure 3). **(A)** One-dimensional cross-section of the luminance profile I(x) of a decremental disk-annulus stimulus. **(B)** The spatial pattern of activation L(x) of lightness neurons in V4 in response to the stimulus in **(A)**. **(C)** The pattern of activation D(x) of darkness neurons in V4 in response to the same stimulus. **(D)** The pattern of activation AC(x) at some subsequent stage of neural processing (likely located in cortical area TEO or TE) at which the activation pattern D(x) is subtracted from L(x) to compute perceived reflectance, or achromatic color. Note that the activation patterns L(x) and D(x) are constructed solely from induction signals based on edges pointing in the direction of the disk center. The rationale for excluding other edge-based induction signals from the computation of disk lightness is discussed later in the paper. Note also that the predicted lightening of the disk center, illustrated as a local peak of activity in the AC(x) activation pattern at the location of the disk center is observed in actual experiments (i.e., Rudd and Zemach, 2004; not previously reported), although the spatial profile of the lightened region has more the form of an inverted meniscus, as would be predicted by a model in which the receptive field profiles of the lightness and darkness neurons in V4 fell off exponentially, rather than linearly, with distance from the receptive field center.

The mechanism by which filling-in and edge integration occur in the present model is further illustrated by Figure 5. The figure shows the responses of the model lightness and darkness neurons whose receptive field centers are located within the interior of a dark disk surrounded by a lighter annulus, as well as the response of a hypothetical mechanism located somewhere beyond V4 that computes the difference of the lightness and darkness neuronal responses at a given location to arrive an overall computation of the achromatic color assigned to that location, which models the observer's percept. As mentioned above, the differencing mechanism is likely located in cortical area TEO or TE, at or near the end of hierarchical ventral processing stream. According to the model, the achromatic color of the disk that is perceived by the observer depends on the difference between the activations of lightness and darkness neurons located with the disk interior. These activations, in turn, depend on the distance and contrast polarity of the lightness- and darkness-inducing edges of the annulus/background and disk/annulus borders. A key difference between the current theory and alternative filling-in theories based on diffusing color signals that diffuse from borders but get stopped at the next border that they encounter is that the lightness induction from the annulus/background border can affect the disk lightness. Furthermore, though the disk appears dark relative to the lighter surround, it is actually computed from a difference of separate darkness and lightness components originating from the disk/annulus and annulus/background borders. In fact, this assumption is needed to explain the quantitative edge integration data modeled by Rudd and Zemach (2004), including the influence on disk lightness of the distance of the annulus/background border from the target disk.

The model in Figure 5 predicts that the disk percept would change dramatically if the influence of either the darkness-inducing disk/annulus edge or lightness-inducing annulus/background edge was removed from the neural computation of achromatic color. If the disk/annulus edge was removed, the stimulus would look like the spatial pattern of activation L(x) of the lightness neuron layer in V4. If the annulus/background edge were removed, the stimulus lightness would look like an upside-down version of the pattern of activation D(x) of the darkness neuron layer in V4. These model predictions are consistent with data from a recent perceptual study by Anstis (2013), who used a flicker masking technique to selectively remove the perceptual influence of either the disk/annulus or annulus/background edge. Masking out the annulus/background edge changed the stimulus percept to that of a small, now darker, disk with no surrounding annulus, whereas masking out the disk/annulus edge changed the percept to one of a large, light disk, whose light achromatic color perceptually filled in across the (now missing) disk/annulus border (see Anstis, 2013, Movie 6). Anstis's results are thus predicted by the neural model on the assumption that flicker masking has the effect of fatiguing the responses of neural edge detector units located in V1 or V2, before lightness and darkness filling-in occur in V4.

**Figure 6:**
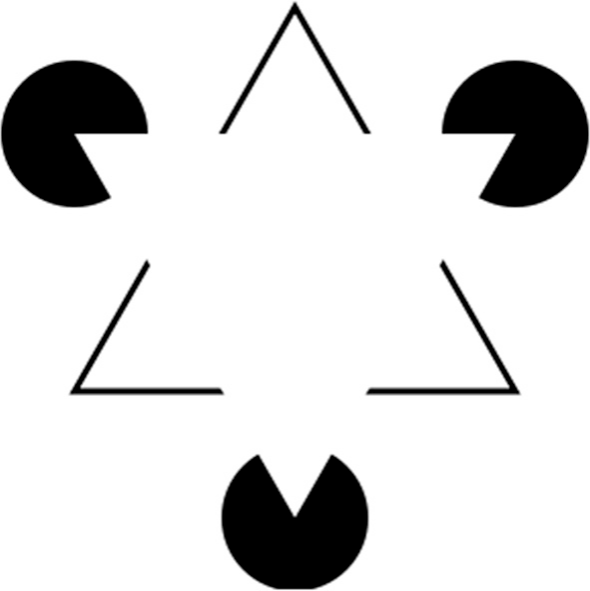
**Kanizsa triangle**. Here, three black pac-men inducers generate in the brain of the observer the percept of an upside-down white (Kanizsa) triangle. The Kanizsa figure appears to partially occlude three black disks and upright triangular outline whose borders appear to complete behind the Kanizsa triangle. The straight edges of the pac-men induce an illusory contour that completes the borders of the Kanizsa figure. The area of the Kanizsa figure appears somewhat whiter than the regions just outside its borders that have the same luminance in the retinal image.

The model shown in Figure 5 cannot, however, explain all aspects of achromatic color filling-in. For example, it cannot explain filling-in phenomena in which regions formed by illusory borders—such as the Kanizsa triangle figure shown in Figure 6 (Kanizsa, 1979)—fill in with a color that is different from the background color: in this case, somewhat lighter. To explain the fact that the Kanizsa triangle appears lighter than the background would require additional neural mechanisms that complete the perceptual borders of the triangle and segment it from the background. But this idea is consistent with the cortical circuit that is here proposed to underlie perceptual filling-in, because neural circuits that support border completion and image segmentation are known to exist in area V2 (von der Heydt et al., 1984), at a stage of cortical processing that comes before the proposed edge integration stage in V4 that performs color filling-in and surface completion, according to the lightness model. So the basic edge integration model could be extended to account for the perceptual filling-in of illusory figures like the Kanizsa triangle by taking these mechanisms into account. I will return later in the paper to the issue of how cortical circuits that perform image segmentation and other functions related to perceptual organization contribute to lightness computation after first discussing the role of visual attention.

## Need for top-down intentional control of edge weights

Arend and Spehar (1993a,b) demonstrated that more than one type of lightness judgment can be performed with simple stimuli comprising a target region embedded in a homogeneous surround. They used computer-generated displays, but instructed their observers to imagine that the stimuli were made of real papers. The luminance of the surround field was varied and the observers were told to imagine that the luminance variation was due either to a change in the reflectance of the surround paper, or to a change in the illumination lighting both the target and surround. Ideally, a change in the surround reflectance should not influence the target lightness. On the other hand, with the target luminance held constant, a change in the perceived intensity of the illumination lighting both the target and its surround should produce a compensatory change in perceived target reflectance, according to the equation L = R × I, where L is retinal luminance, R reflectance, and I illumination. In the actual experiment, the different instructions produced lightness judgments roughly in accord with these two ideal observer models.

**Figure 7:**
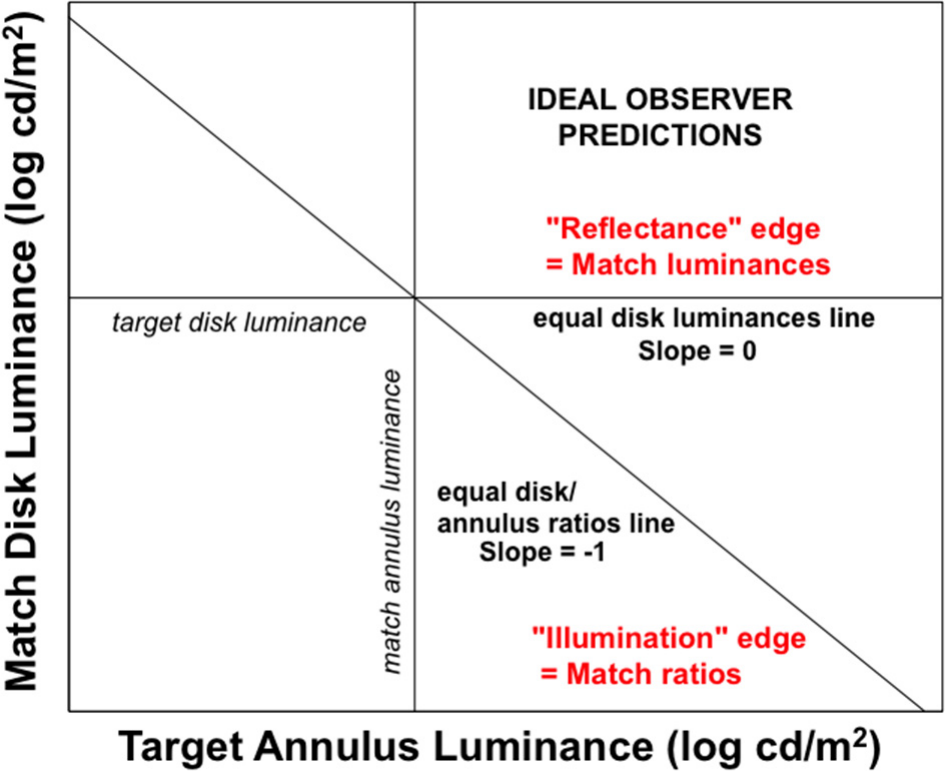
**Ideal observer predictions for lightness matching in a disk-annulus display under two different assumptions about the nature of the illuminant**. When the luminance step at the outer edge of the annulus is judged to be the result of a change in surface reflectance, it will ideally contribute to an edge integration computation of lightness (perceived reflectance). The goal of such a computation is to veridically relate the target disk reflectance to the reflectance of the background field and, by extension, to the reflectance of another, matching, disk, viewed against the same background. The computation is carried out by spatially integrating the two luminance steps. Since no illumination variation is assumed, both disks are implicitly assumed to be viewed under the same illumination conditions and a reflectance match corresponds to a luminance match. When the luminance step at the outer edge of the annulus is judged to be the result of an illumination change, on the other hand, the luminance step at the outer annulus edge should *not* be included in a computation that seeks to relate the disk reflectance to that of the background field. In this case, the “annulus” is interpreted to be a differently illuminated region of the background field and not a separate surface with a different reflectance, The disk lightness can thus be related to the background by computing the disk/annulus luminance ratio—in log units, the step in log luminance at the disk/annulus edge, alone. The luminance matching and ratio matching predictions corresponding to the two interpretations in which the outer annulus edge is either seen as a reflectance edge or illumination edge are indicated by straight lines with slopes of 0 and -1, respectively, on this log-log plot.

**Figure 8:**
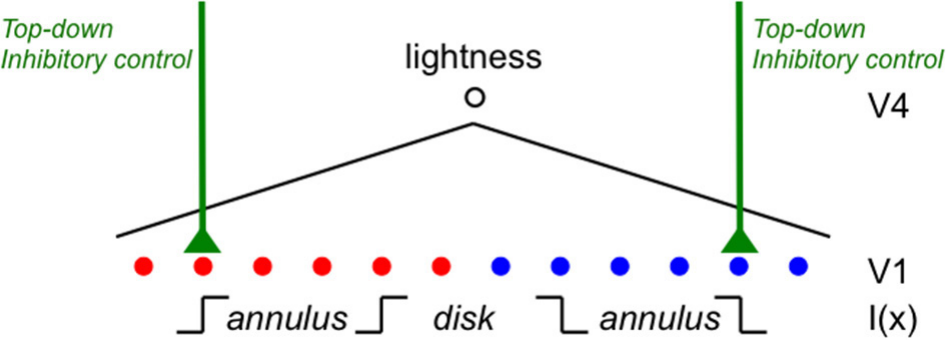
**A cortical mechanism that utilizes top-down feedback to perform different lightness judgments given different assumptions about the illumination with the same disk-annulus stimulus**. The proposed cortical circuit by which the luminance step at the outer annulus edge in an incremental disk-annulus display is either included or excluded from the computation of the disk lightness to instantiate either of the two ideal observer models whose behavior is illustrated in Figure 7. When the outer annulus edge is interpreted as an illumination edge, the responses of edge detector neurons in V1 are suppressed by a top-down neural gain control. This inhibitory gain control may originate from prefrontal cortex and act either directly on V1 neurons—by shutting down cortical columns containing neurons that encode edges tuned to a particular orientation and contrast polarity, and located in the parts of the visual field corresponding to the outer edges of the annulus—or indirectly via IT (see text for further details). Similar inhibitory connections are assumed to project to all edge sensitive neurons in V1, but only the connections that are relevant to the lightness matching task for incremental disk-annulus stimuli are shown.

Rudd (2010) quantified the actual lightness matches made under these different lightness matching instructions using side-by-side target and matching disk-annulus stimuli displayed on a computer monitor. The luminance of the right annulus was experimentally varied and the observer adjusted the left disk luminance to match the right disk in lightness. The ideal observer predictions for the two instruction sets are associated with different ideal edge weight settings in the edge integration model (Rudd, 2010). When an observer is instructed to interpret a change in the annulus luminance as a reflectance change, the outer and inner edges of the annulus should both be interpreted as reflectance edges and therefore both should contribute to the perceptual representation of the disk reflectance. The inner edge provides information about the reflectance ratio between the disk and annulus; and the outer edge provides information about the reflectance ratio between the annulus and the background. If this information is known on both sides of the display—and if it represented veridically by the visual system—then the reflectances of the two disks can be accurately compared by integrating the steps on log reflectance from the background field to each target disk. If, on the other hand, the observer is instructed to interpret changes in the annulus luminance as signifying changes in the illumination lighting both the disk and annulus on that side of the display, then the outer edge of the annulus should be interpreted as an illumination edge and it should not contribute to a calculation that attempts to relate the disk reflectance to the reflectance of the background field. Only the luminance ratio at the disk/annulus edge should contribute to that calculation. Figure 7 illustrates the predictions of the ideal observer models for the case in which the outer edge of the annulus is a reflectance edge and the case in which the outer annulus edge is an illumination edge. Figure 8 shows how the computations corresponding to each situation could be performed with the proposed cortical edge integration mechanism through the strategic control of top-down inhibitory feedback to neural edge detectors in V1. Note that this inhibition, when active, eliminates from the edge integration computations the luminance step corresponding to the annulus/background edge (because it is assumed to be an illumination edge, rather than a reflectance edge). This impacts the response of the lightness model in the same way that masking the outer annulus border in Anstis's contour adaptation experiment does.

The actual lightness matches made under the two different sets of assumptions about the correct interpretation of the luminance step at the outer annulus border were approximately consistent with these ideal observer predictions. When given the “change in reflectance” instructions, the luminance ratios at the inner and outer annulus edges both contributed to the observers' lightness matches, in roughly equal proportion, as would be expected if both borders were interpreted as reflectance edges. When given the “change in illuminance” instructions, the observers placed considerably more weight on the luminance ratio at the disk/annulus edge, but the outer annulus edge also made a contribution to the disk lightness, consistent with the idea that this border was interpreted as likely being due to a change in illumination. Since the physical stimulus was identical in the two instruction conditions, any changes in the perceptual weight given to the outer annulus edge must have been due to a top-down, task-specific, influence on the edge weights. If, as hypothesized, these weights are manipulated by neural gain control applied to simple cell responses in V1, then it must be the case that observers can utilize top-down gain modulation to V1 to reconfigure the results of the subsequent neural lightness computations at later stages of ventral stream processing. The observer's intentional control of top-down feedback to carry out these instruction-specific lightness judgments might, in principle, be instantiated by direct connections from the frontal lobe that have been recently demonstrated to be capable of shutting down the gain of cortical columns in V1 (Massimo Scanziani, talk given at the University of Washington, Department of Physiology and Biophysics, October, 2012) or by feedback projections from prefrontal cortex to V1 that are relayed through inferotemporal cortex (Gilbert and Li, 2013).

## Influence of figure-ground segregation on lightness and mid-level computations in V2

Gelb (1929) showed that a single achromatic paper illuminated by an intense spotlight appears white, regardless of the paper's actual gray level. A charcoal paper viewed in isolation in the spotlight will look just as white as will a mid-gray paper or an actual white paper. However, if a gray paper is added to a spotlight already containing a charcoal paper, the gray paper will now appear white and the charcoal paper will appear darker by comparison. If a third, actual white, paper is added to the spotlight, the appearances of the gray and charcoal papers will be further darkened. This effect has been explained by Gilchrist (2006; Gilchrist et al., 1999) as a consequence of highest luminance anchoring: the surface with the highest luminance in the spotlight appears white and the lightnesses of the other surfaces in the spotlight are scaled relative to the white point. Rudd and Zemach (2005; Rudd, 2013; Kingdom, 2011) proposed an alternative anchoring rule—highest *reflectance* anchoring—that can also account for the effect. The meaning of highest reflectance anchoring is that the highest reflectance in the scene always appears white, but the highest reflectance may not always be the same as the highest luminance due to lightness induction exerted by a lower-luminance context, which can differ for different highest luminance patches, as shown by Rudd and Zemach (see also Rudd, 2013). A recent study of the Gelb effect, Vladusich (2013a) found that such lightness induction from lower luminance contextual elements can change the appearance of even the region with the highest *lightness*, a result that may call both of these anchoring rules into question. In the absence of any type of anchoring, the edge integration theory described below would predict that lightness of the Gelb papers would grow in proportion to the cube root of reflectance.

Cataliotti and Gilchrist (1995) measured the individual lightnesses of a series of achromatic papers shown together in such a spotlight, an experimental paradigm known as the “staircase-Gelb paradigm” (Gilchrist et al., 1999; Gilchrist, 2006). They discovered the scale of paper lightnesses to be strongly compressed relative to their actual physical reflectance scale. Rudd (2013) re-analyzed their data and showed that the lightness scale is related to scale of the paper luminances in the observer's image of the scene by an equation having the same form as Stevens' brightness law, only here lightness—rather than brightness—is related to luminance by a power law of exponent 1/3. This finding again argues that lightness and brightness perception share some common underlying neural substrates. In another experiment, Cataliotti and Gilchrist discovered that surrounding the Gelb papers with a white frame greatly alters the lightness scaling of the papers, an effect that they named *insulation*. With the white frame present, lightness varies as a linear function of both reflectance and luminance; in other words, lightness varies as a power law of luminance in which the power law exponent is 1, rather than 1/3.

**Figure 9:**
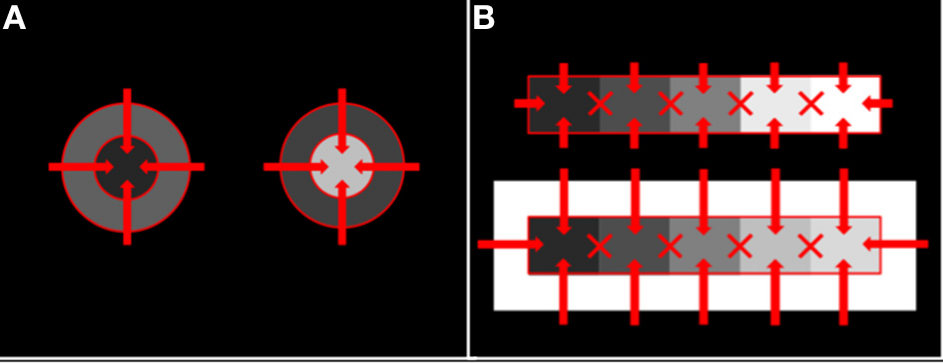
**Allowed and unallowed edge integration paths in disk-annulus and staircase-Gelb lightness paradigms. (A)** Disk-annulus. **(B)** Staircase-Gelb. For both types of stimuli, edge integration takes place only along direct paths from the common background, or surround, field to the target whose lightness is begin computed. Edges whose weighted log luminance ratios are integrated to compute target lightness are indicated by solid red lines. The X's indicate edges that do not participate in edge integration.

Anchoring theory cannot explain either the compression or insulation effect. To explain these, Rudd (2013) elaborated the neural edge integration theory described above. The elaborated model posits that the only edges that participate in the edge integration process in V4 are edges that lie along a direct path from the common background to the target whose lightness is being computed. In the case of the staircase-Gelb stimulus, the relevant edges are those forming the interface between each paper and the dark surround or white frame. The borders between individual Gelb papers are excluded from participating in edge integration (Figure 9).

Unlike anchoring theory, Rudd's elaborated edge integration theory assumes that lightness is computed by comparing each target's luminance to the same common *background field*, rather than to the highest luminance surface appearing within the target's illumination framework. Steps in log luminance along the path from the common background to the target paper are spatially summed to place the target on the same lightness scale as the rest of the Gelb papers, whose luminances are also compared to the common background region to compute their lightnesses. Perceptual distortions, such as compression, may be introduced in the process of mapping the physical reflectance scale to a common lightness scale. Distortions arise when the weights assigned to edges are not equal to 1, which occurs in the model because of the contrast polarity- and distance-dependency of the edge weightings. Importantly, the elaborated theory is consistent in terms of the edge-weighting principles that it uses to account for the quantitative magnitudes of the lightness illusions produced in the Gelb effect, simultaneous contrast, disk-annulus lightness paradigms (Rudd, 2013).

If edge integration only occurs along paths from the common background to the target, then the brain must have some special circuitry that perceptually organizes the image into figure and ground, and excludes some edges from participating in edge integration. This circuitry must lie somewhere along the path from the edge encoding neurons in V1 to the long-range edge integration mechanism in V4. Consistent with this requirement, von der Heydt and his colleagues (Zhou et al., 2000; von der Heydt et al., 2003) have demonstrated the existence of “border ownership” neurons in V2 that have the right properties to distinguish between edges that separate figural from background regions and edges that do not. Such neurons respond only to the borders belonging to a closed figure. Border ownership neurons appear to derive their properties through feedback interaction with higher-level pattern recognition networks in the inferotemporal cortex (IT) (Craft et al., 2007). In principle, these networks might be able to differentiate between the border surrounding the entire series of papers in a staircase-Gelb display—that is, the border between the papers and the common background—and the borders surrounding each individual paper. If so, they could form the basis for an edge integration computation that gives nonzero weights only to borders lying along a path between a common background and target regions (that is, the border surrounding the entire set of papers). In the following section, I further elaborate this idea to suggest that the outputs of border ownership circuits in V2 can be modulated by top-down attentional feedback to give nonzero weights to *either* the local borders of a Gelb paper or the larger border separating the Gelb series as a whole from the common background. Because such a mechanism is required by the model but has not yet been demonstrated, the existence of a cortical mechanism by which attentional modulation can select either the larger border surrounding the entire set of Gelb papers, or the local border surrounding an individual paper, as the target border for a border ownership signal in V2 is a prediction of the model.

## Top-down modulation of edge weights and individual differences in lightness

Rudd and Zemach (2005) performed a lightness matching study using the same disk-annulus displays that were later used in the study by Rudd (2010) but without giving their observers any special instructions regarding how to interpret changes in the annulus luminance. The lightness matches varied considerably across the three observers in the study. One showed almost no lightness induction, while the other two exhibited induction effects of moderate size. In light of the later findings from the 2010 study, it seems likely that the wide range of inter-observer differences in the 2005 study resulted from different interpretations of meaning of the change in annulus luminance.

The neural edge integration theory proposed here attributes these individual differences to changes in the strength of the top-down gain control applied to the outer edge of the annulus. As discussed above, when an observer interprets the outer annulus edge as an illumination edge, he should ideally exclude this edge from his computation of disk lightness because an illumination edge is uninformative with respect computing a map of the relative reflectances of surfaces within the visual scene (Rudd, 2013). He should therefore set the weight given to that edge to zero and base his disk lightness judgments strictly on the luminance ratio between the disk and annulus because this is the only ratio that signals a reflectance change along path from the common background of the two disk-annulus patterns in the display and the target disk whose lightness was being computed. If, on the other hand, the outer annulus edge is interpreted to be a reflectance edge, then it should make a large a contribution to the calculations of the disk luminance as the inner annulus edge.

In this special case of disk-annulus displays, the outer annulus edge could either be included or excluded from the disk lightness computation simply by varying the size of a spotlight of attention, which could be realized by a top-down attentional gain field. A small spotlight would assign a gain of 1 to V1 neurons responding to the edge between the disk and annulus, and a gain of 0 to the edge between the annulus and background field. A large spotlight would assign a gain of 1 to both edges. In the edge integration theory of lightness judgments in the Gelb paradigm proposed in Rudd (2013), it was posited that only the common border between the Gelb papers and the background that surrounds the entire set of papers participates in the computation of each paper's lightness. This is equivalent to saying that the observer compares each paper to the same common background field in order to compute the paper's relative reflectance. Since all of the papers are compared to the same background field, the perceived reflectances of the Gelb papers will be placed on the same perceptual scale.

**Figure 10:**
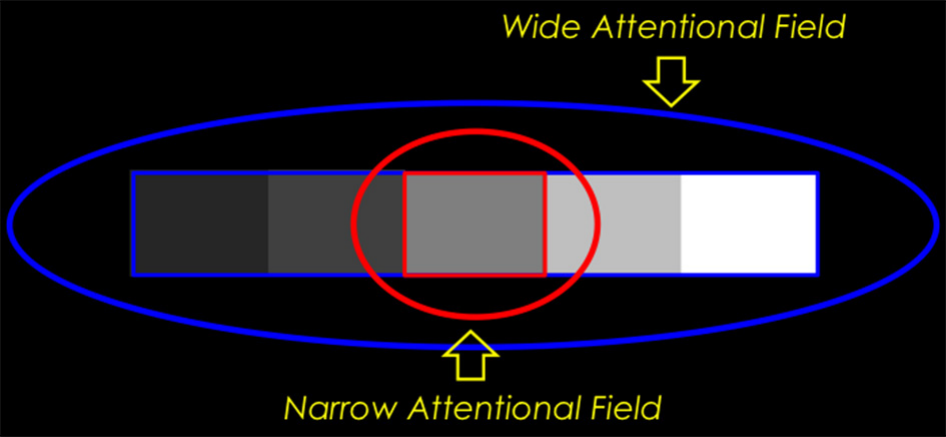
**Narrow-field vs. wide-field attentional processing of the paper lightnesses in the staircase-Gelb experiment**. The observer suppresses through top-down feedback to V1 the responses of simple cells lying outside his field of spatial attention. When the observer examines the Gelb papers as a gestalt (attentional spotlight = blue oval), border ownership circuits in his cortical area V2 identify the border surrounding all five Gelb papers (blue rectangle); lightness is computed by comparing the luminance of each paper to the luminance of the common background field. When the observer focuses his attention on an individual paper (red oval), his border ownership circuits identify the border of that paper only (red rectangle); the paper lightness is computed by averaging the log luminance ratios of the four borders of the paper.

Alternatively, we might imagine that the observers in the staircase-Gelb experiment differ in the way they judge the paper lightnesses. An observer with a narrow attentional spotlight might focus his spotlight on a single Gelb paper, comparing that paper only to the immediately adjacent image regions. An observer with a large attentional spotlight, on the other hand, might take the entire set of papers into account, comparing each paper to the same common background, as proposed by Rudd (2013). These alternative perceptual analyses are illustrated in Figure 10.

According to edge integration theory, a narrow attentional spotlight would mean that only the immediate borders of the target paper would survive the top-down gain modulation of simple cell responses in V1. The border ownership computation in V2 therefore would not have the full border surrounding the entire set of Gelb papers to operate on. The immediate borders of the target paper could potentially be identified as forming a closed border, but the larger border surrounding the set of papers could not. A border ownership circuit in V2 would therefore not be able to discover the figure-ground relation that segregates the set of papers from the background. It follows that the only edges that would be input to edge integration computation in V4 for the purpose of computing the target's lightness would be the immediate edges of the target. The lightness computed by the narrow spotlight observer would be based on a sum of the luminance steps (in log units) computed at the four edges of the target paper.

In the case of a wide attentional spotlight—one large enough to include the entire series of Gelb papers—the border ownership computation in V2 *would* have the full border surrounding the Gelb papers to operate on. In theory, neural circuits involving V2 could identify this border as a border that surrounds an “object” consisting of the whole Gelb paper series. According to the edge integration model, only this border will then contribute to the computation of each paper's lightness. Thus, for a wide-field attender, the lightness of any given paper will be determined entirely by the luminance ratio at the borders between the paper and the common surround in which the larger object consisting of the entire series of papers Gelb is embedded. This is the situation that I showed earlier leads to an explanation of the 1/3 power law compression that characterizes the Cataliotti and Gilchrist data. Thus, the Cataliotti and Gilchrist results are explained on the basis of an edge integration model in which the observer is assumed to have a wide attentional spotlight.

A primary motivation for anchoring theory was the lack of evidence in Cataliotti and Gilchrist's study for any influence on target lightness of the papers that neighbored it. They demonstrated the absence of such an effect by showing that scrambling the order of the Gelb papers made no difference to lightness matches made to the individual papers; only the highest luminance paper within the spotlight influenced the target matches. Again, this is consistent with the edge integration prediction for wide-field observers. But other experimenters have replicated their study and found spatial ordering effects (Zavagno et al., 2004; Blakeslee et al., 2009). The spatial ordering effects contradict anchoring theory and therefore have been a source of controversy. Edge integration theory can explain the differences in the results obtained across studies as effects of individual differences if it additionally assumed that the studies demonstrating spatial ordering effects were based on narrow-field observers, while the Cataliotti and Gilchrist results were based on based on wide-field observers. It is unclear whether the stimulus conditions in the different studies varied in ways that might have caused the observers to adopt either a narrow- or wide-field attentional spotlight. A full analysis of the differences between experiments and the test of the model for narrow-field observers against the data from the studies that yielded the scrambling effect are left for an upcoming paper. Here, I simply note that intermediate spotlight sizes might also be allowed, so a full analysis should consider whether any spotlight size can explain the Gelb matching data from studies in which a scrambling effect was obtained, under the constraint that the weights given to positive steps in log luminance are always 1/3 and the weights given to negative steps in log luminance are always 1, after controlling for the effect of distance from the target.

## Some outstanding problems for the theory

**Figure 11:**
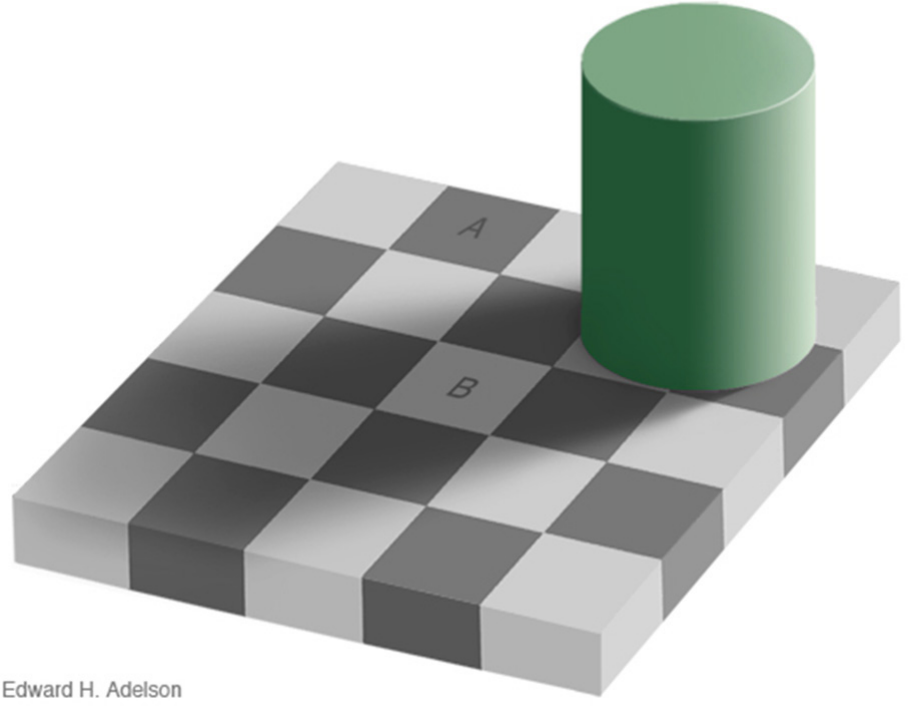
**Adelson checker-shadow figure**. A and B have the same luminance but A appears as a brightly-lit dark surface, while B appears as a light surface viewed in shadow. Copyright 1995, Edward H. Adelson. Used by permission; http://persci.mit.edu/gallery/checkershadow.

In the natural world, observers are not usually given instructions concerning whether to interpret spatial luminance variations as the result of either reflectance or illumination variation; the process is largely carried out at an unconscious level (von Helmholtz, 1866/1924). The checker-shadow figure of Adelson (Figure 11) illustrates the basic problem that the visual system confronts in trying to differentiate the separate causal effects of reflectance and illumination in producing the retinal image. Here, checks A and B have the same luminance, but A appears to be highly illuminated surface with low reflectance, while B is seen as a white surface viewed in shadow.

One perceptual cue that may help differentiate reflectance from illumination variation in Adelson's figure is the sharpness of the spatial luminance change. The reflectance edges in the figure are all “hard” edges, while the illumination edge is a gradient. The purpose of edge integration is to map the surfaces within the image to a common scale of perceived *reflectance* (Rudd, 2013). For the purpose of computing relative reflectance, luminance steps or gradients in the input image caused by illumination variation should ideally be left out of the edge integration computation. If an edge integration computation left out the change in luminance across the gradient, it would effectively “discount the illuminant” and correctly estimate the relative reflectances of the surfaces depicted in the figure. Such a computation could account, at least qualitatively, for the perceived lightness difference between checks A and B. An edge integration path from A to B, for example, would not integrate the luminance gradient produced by the shadow. Thus, check B would appear “lighter” than it would if the edge integration process integrated all luminance steps. If on the other hand, all steps in luminance, including those associated with gradients, were integrated perfectly, then checks A and B would have the same appearance. So it is clear that the visual system does not integrate all of the luminance steps.

Rudd (2010, 2013; see also Rudd and Popa, 2007) pointed out that a neural mechanism that log transforms the pointwise luminances in the image prior to spatial filtering by Gabor-like simple cell receptive fields in V1 would produce simple cell responses that encode logarithms of luminance *ratios* at different orientations over a range of spatial frequencies. Only low-spatial frequency sensitive simple cells (i.e., large scale Gabor filters) would transmit information about the presence of the illumination gradient in Figure 11. Since the check edges contain information at high spatial frequencies, reflectance edges could potentially be distinguished from illumination gradients on the basis of spatial frequency. Thus, the perceived lightness of the checks could be explained by a version of the edge integration model in which the outputs of low spatial frequency-sensitive simple cells in V1 are excluded from the long-range spatial edge integration in V4. This hypothesis is consistent with the fact that the perceptual effects of the shadow in Figure 11 cannot be intentionally “undone” by the observer. That is, it is impossible to make checks A and B look the same, even if you try. The case of the checker-shadow figure is thus fundamentally different than case of the instructional effects studied by Rudd (2010), in which the outer annulus edge in a disk-annulus display could be consciously interpreted as resulting from either a reflectance edge or an illumination change, and lightness judgments in line with these alternatively interpretations made interchangeably. Both cases might be explained if top-down feedback can either allow or suppress high spatial frequency image content from entering into the edge integration process; whereas, low spatial frequency content is automatically given smaller perceptual weight, or perhaps excluded entirely, from the edge integration that occurs at a subsequent stage of processing in V4.

If this explanation of the checker-shadow percept is correct, then it raises the question of how we can even *see* the illumination gradient in the figure. One possibility is that the edge integration the circuit in V4 is specialized to represent relative reflectance *independent* of illumination, rather than to represent both reflectance and illumination. This interpretation would appear to be consistent with studies of clinical patients with damage to V4, who exhibit deficits in color constancy while retaining their ability to make local luminance and wavelength discriminations that could be performed by neurons in V1 (Zeki and Bartels, 1999). Alternatively, V4 might receive input from *either* low- *or* high-spatial frequency-tuned neurons—both not both at once—for the purpose of processing reflectance or illumination, respectively. This second hypothesis would be consistent with evidence that diagnosticity changes the perception of stimuli containing content at multiple spatial scales (Oliva and Schyns, 1997). These alternative hypotheses raise important additional questions both with respect to the nature of the neural mechanisms supporting lightness and the locus or loci of the neural correlates of conscious visual perception that should be explored in future research.

## Comparison with gamut relativity theory: transparency and “dimensions” of lightness

Vladusich (2013a) recently proposed an alternative theory of lightness computation—gamut relativity theory—that addresses some of the same issues and data addressed in the present paper. The two theories converge in some ways, but differ fundamentally in others. A significant point of convergence is the assumption—now supported by a considerable amount of data—that darkness induction is quantitatively stronger than lightness induction. The existence of an asymmetry in the strengths of lightness and darkness induction has long been recognized by psychophysicists (Wallach, 1963; Heinemann, 1972; Gilchrist, 2006), but the idea that this asymmetry is quantitative, rather than absolute, is recent (Rudd and Zemach, 2004, 2005, 2007; Rudd, 2013; Vladusich, 2013a,b). Edge integration theory and gamut relativity theory agree that darkness induction is quantitatively stronger than lightness induction. However, the two theories differ in their estimates of the specific degree of quantitative asymmetry between light and darkness induction. Vladusich (2013b) derived a lightness law exponent of 1/2 from theoretical principles and modeled reflectance matching in the staircase-Gelb and related paradigms on the basis of this assumption (Vladusich, 2013b). The present model assumes a lightness law exponent of 1/3 on the basis of Stevens' power law for brightness (of increments) (Stevens, 1975), as well as both direct physiological evidence of a 1/3 power law compression in V1 (Kay et al., 2013) and indirect evidence for a 1/3 power law transformation of incremental luminance from studies of simple reaction time (Pieron, 1914; Luce, 1986) and critical duration (Raab, 1962; Rudd, 1996). The fact that an asymmetry in the magnitudes of neural ON and OFF responses is seen even in the responses of cone photoreceptors (Angueyra and Rieke, 2013) further reinforces the idea that the lightness-darkness asymmetry originates from one or more neural transformations of the physical stimulus occurring early in the stream of visual processing.

**Figure 12:**
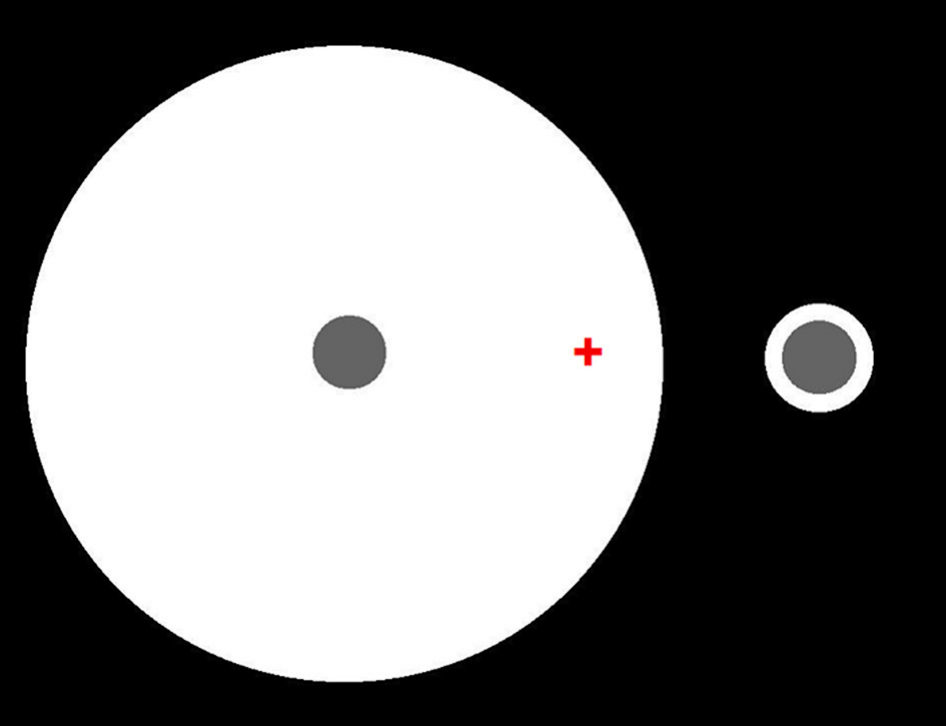
**Identical decremental disks seen in the context of annular surrounds having the same luminance but different widths**. Fixate on the red cross. The left disk should appear darker than the right disk, even though they have the same luminance. Edge integration theory accounts for the different disk lightnesses by asserting that the disk lightness is computed from a weighted sum of the directed steps in log luminance at the disk/annulus and annulus/background borders. Thus, the disk lightness does not just depend on luminance or local contrast, but on a computation that is carried out over an extended region of the scene. Because luminance step at the disk/annulus border decreases in the direction of the disk, that border tends to darken the disk appearance. Because luminance step at the disk/annulus border increases in the direction of the disk, that border tends to lighten the disk appearance. The quantitative contribution of the disk/annulus border to the total disk lightness is the same for the two disks because the size and direction of the luminance step is the same in the two cases, and the border is in the same location relative to the disk center. The quantitative contribution of the annulus/background border to the total disk lightness is smaller in the case of the large annulus because the annulus background edge is further from the disk in that case, even though the luminance steps have the same magnitude and direction.

Edge integration theory and gamut relativity theory also differ in other, more important, ways. Perhaps most significantly, they differ with respect to the status of the spatial integration of spatially oriented lightness and darkness induction signals. Gamut relativity theory models lightness and darkness as separate dimensions of achromatic color, which remain distinct up to the level of human awareness (Vladusich et al., 2007; Vladusich, 2013a). Edge integration theory, on the other hand, assumes that positive and negative luminance steps can be neurally bound to form a single dimensions of surface reflectance. Figure 12 presents a simple demonstration that refutes the idea that human vision encodes lightness and darkness as separate dimensions. Here, two identical disks are surrounded by annuli having the same luminance, but different width. The disks differ in lightness, which clearly shows that percepts of darkness depend on factors other than the magnitude of decremental luminance: namely, effects of spatial context. Edge integration theory explains this phenomenon on the assumption that the disk appearance is computed by a weighted sum of the decremental luminance step at the disk edge and the incremental luminance step at the outer annulus edge: in other words, by a binding together of darkness and lightness signals to neurally represent the disk reflectance.

It should perhaps be emphasized that I do not claim that luminance steps are always bound together to compute lightness. For instance, the visual system tries to exclude from the edge integration process any image steps in log luminance resulting from spatial changes in illumination. Rudd (2013) discusses why the integration of reflectance edges is essential for establishing a unitary scale of surface reflectance that applies to surfaces viewed under a common illumination. In the absence of such a scale, there would no meaningful perceptual estimate of reflectance; the lightness of individual surfaces could only be judged relative to the luminance of their local surround elements. Relatedly, an edge integration algorithm that is designed to achieve constancy will only be successful if it filters out image luminance steps resulting from illumination variation. This does not mean that the illumination field is not also neurally represented; only that the visual system must perform edge classification prior to building separate representations of and reflectance illumination. Clearly, the observer can perceptually access either the reflectance representation or the illumination representation, though they remain categorically distinct in perception.

Another important case in which edges are combined to produce a unitary reflectance scale is that of perceptual transparency. One important factor that contributes to the perception of transparent layers is edge contrast polarity (Adelson, 2000; Roncato and Casco, 2003). But transparent layering cannot be explained solely by sorting layers based on contrast polarity. Figural properties are also critical in the construction of transparency perceptual layers (e.g., Adelson, 2000; Anderson, 2003; Anderson and Winawer, 2005). In ecological vision, the process of segmenting the filter from the background is aided by depth cues, such as binocular disparity and motion parallax. What is needed to account for transparency is a theory that can explain why light and dark elements sometimes group together to form unitary lightness scales, and sometimes do not. Clearly, this is a challenging problem. From the neural standpoint, the solution requires the input of cortical image segmentation mechanisms. Although I have briefly discussed such mechanisms here, the goal of the present work is not to describe how such mechanisms work at the algorithmic level, but rather to an outline model of how they fit into the larger cortical circuit that computes lightness.

### Conflict of interest statement

The author declares that the research was conducted in the absence of any commercial or financial relationships that could be construed as a potential conflict of interest.
